# COVID-19 in Cancer Patients, Risk Factors for Disease and Adverse Outcome, a Population-Based Study From Norway

**DOI:** 10.3389/fonc.2021.652535

**Published:** 2021-03-25

**Authors:** Tom Børge Johannesen, Sigbjørn Smeland, Stein Aaserud, Eirik Alnes Buanes, Anna Skog, Giske Ursin, Åslaug Helland

**Affiliations:** ^1^ Registry Department, Cancer Registry of Norway, Oslo, Norway; ^2^ Institute of Clinical Medicine, University of Oslo, Oslo, Norway; ^3^ Division of Cancer Medicine, Oslo University Hospital, Oslo, Norway; ^4^ Norwegian Intensive Care and Pandemic Registry (NIPaR), Bergen Health Trust, Bergen, Norway; ^5^ Department of Anaesthesiology and Intensive Care, Haukeland University Hospital, Bergen, Norway; ^6^ Institute of Basic Medical Sciences, University of Oslo, Oslo, Norway; ^7^ Department of Preventive Medicine, University of Southern California, Los Angeles, CA, United States; ^8^ Department of Genetics, Institute for Cancer Research, Norwegian Radium Hospital, Oslo University Hospital, Oslo, Norway

**Keywords:** cancer, COVID-19, population-based, death, intensive and critical care, cancer treatment

## Abstract

**Background:**

Cancer has been suggested as a risk factor for severe outcome of SARS-CoV-2 infection. In this population-based study we aimed to identify factors associated with higher risk of COVID-19 and adverse outcome.

**Methods:**

Data on all confirmed SARS-CoV-2 positive patients in the period January 1 to May 31, 2020 were extracted from the Norwegian Surveillance System for Communicable Diseases. Data on cancer and treatment was available from the Cancer Registry of Norway, the Norwegian Patient Registry and the Norwegian Prescription Database. Deaths due to COVID-19 were extracted from the Cause of Death Registry. From the Norwegian Intensive Care and Pandemic Registry we retrieved data on admittance to hospital and intensive care. We determined rates of COVID-19 disease in cancer patients and the rest of the population. We also ran multivariate analyses adjusting for age and gender.

**Results:**

A total of 8 410 patients were diagnosed with SARS-CoV-2 infection in Norway during the study period, of which 547 (6.5%) were cancer patients. Overall, we found similar age adjusted rates of COVID-19 in the population with cancer as in the population without cancer. Unadjusted analysis showed that patients having undergone major surgery within the past 3 months had an increased risk of COVID-19 while we did not find increased Odds Ratio (OR) related to other oncological treatment modalities. No patients treated with stem cell or bone marrow transplant were diagnosed with COVID-19. The fatality rate of COVID-19 among cancer patients was 0.10. This was similar to non-cancer patients, when adjusting for age and sex with OR (95% CI) for death= 0.99 (0.68–1.42). Patients with distant metastases had significantly increased OR of death due to COVID-19 disease of 9.31 (95% CI 2.60–33.34). For the combined outcome death and/or admittance to hospital due to COVID-19, we found significant two-fold increased risk estimates for patients diagnosed with cancer less than one 1 year ago (OR 2.08, 95% CI 1.14–3.80), for those treated with anti-cancer drugs during the past 3 months (OR 1.80, 95% CI 1.07–3.01) and for patients undergoing major surgery during the past 3 months (OR 2.19, 95% CI 1.40–3.44).

## Introduction

Worldwide more than 113 million confirmed cases of COVID-19 and more than 2.5 million deaths have been registered by March 1, 2021 (1). Norway has experienced comparatively low numbers of confirmed cases. An adverse outcome of a SARS-CoV-2 infection is associated with known risk factors such as high age and comorbidities including cancer (2–4). The risk of a serious outcome among cancer patients with COVID-19 disease has been less clear.

Some reports have indicated that among cancer patients, especially male gender and hematological malignancies appear to increase the risk of contracting a SARS-CoV-2 infection (5, 6). Elderly cancer patients and those with chronic illnesses and compromised immune system are reported to be most at risk of hospitalization, treatment at intensive care unit (ICU) or death due to COVID-19 (7–9). An early report on the risk of death in patients with COVID-19 disease showed that diseases such as hypertension, diabetes, cardiovascular disease, respiratory disease and cancer were associated with increased risk of death (10). Studies report that among cancer patients with COVID-19, 21% died, compared to 7.8% in non-cancer groups (11, 12). On the other hand, a study from United Kingdom including 16 749 hospitalized patients, reported only a modest but significant increased risk of death for patients with cancer with a hazard ratio of 1.13 (95% CI 1.02-1.24) (13).

A UK cohort of 17 million adults showed that among patients with COVID-19, patients with hematologic malignancies were at higher risk of dying, irrespective of time since diagnosis, while patients with solid tumors were at increased risk of dying if diagnosed the past 5 years (9). The UK coronavirus monitoring project examined 1 044 individuals with cancer and COVID-19 (14) and found that patients with leukemia had a significantly higher risk of COVID-19 related death. This is in contrast to data from a US report on a cohort of cancer patients reporting no increased risk of dying for leukemia patients (15). It has been argued that longer intervals between a cancer diagnosis and the SARS-CoV-2 infection (1–5 years or >5 years) may reduce risk of COVID-19 severity and death compared to patients with a recent cancer diagnosis (< 1 year) (16). Recent or current chemotherapy in patients with hematological malignancies has been found to increase mortality risk (14, 16). Studies of patients with solid tumors have not shown a significant excess mortality risk for recent chemotherapy (14, 15). An analysis from Sweden of COVID-19 positive patients showed that cancer patients who had undergone chemotherapy the past 90 days had a threefold increased risk of death from COVID-19. Further, patients diagnosed with cancer in 2019 or 2020 were at increased risk of death, especially patients with lung and hematological cancers (17).

A multinational study from the US, Canada and Spain of 1 035 cancer patients with COVID-19 diseases found that cancer patients were at increased risk of admission to a hospital, admission to an ICU and need of mechanical ventilation, regardless of cancer type or anticancer treatment (15). Patients with active cancer had a worse outcome.

One meta-analysis reported that risk of severe disease was 45.4% in cancer patients, the risk of being admitted to an ICU was 14.5%, and the risk of requiring mechanical ventilation was 11.7% yielding odds ratios (ORs) between 3 and 4 for all outcomes (11).

To summarize, most studies of cancer patients with COVID-19 have been single-center studies and there is heterogeneity in inclusion and results. A weakness is that several of these studies have been case series, and it is difficult to conclude how the findings are applicable to a more general population. Further, comparisons across populations are difficult with different inclusion criteria. Thus, population based data of high quality comparing patients with and without cancer are limited.

Cancer patients represent a heterogeneous group, and additional knowledge is needed on which patients and which tumor- and treatment related factors confer an increased risk for infection and adverse outcome in order to understand whether an increased COVID-19 risk should impact cancer treatment (18).

The objective of this study was to determine the prevalence and the risk of severe COVID-19 infection among cancer patients compared to the general population. In addition, we aimed to determine risk of severe COVID-19 disease in subgroups of cancer patients based on tumor characteristics and treatment related factors.

## Methods

This study was a population-based retrospective registry study where we compared occurrence and severity of COVID-19 disease in the first few months of the pandemic between cancer patients and the population without cancer.

**Figure 1:**
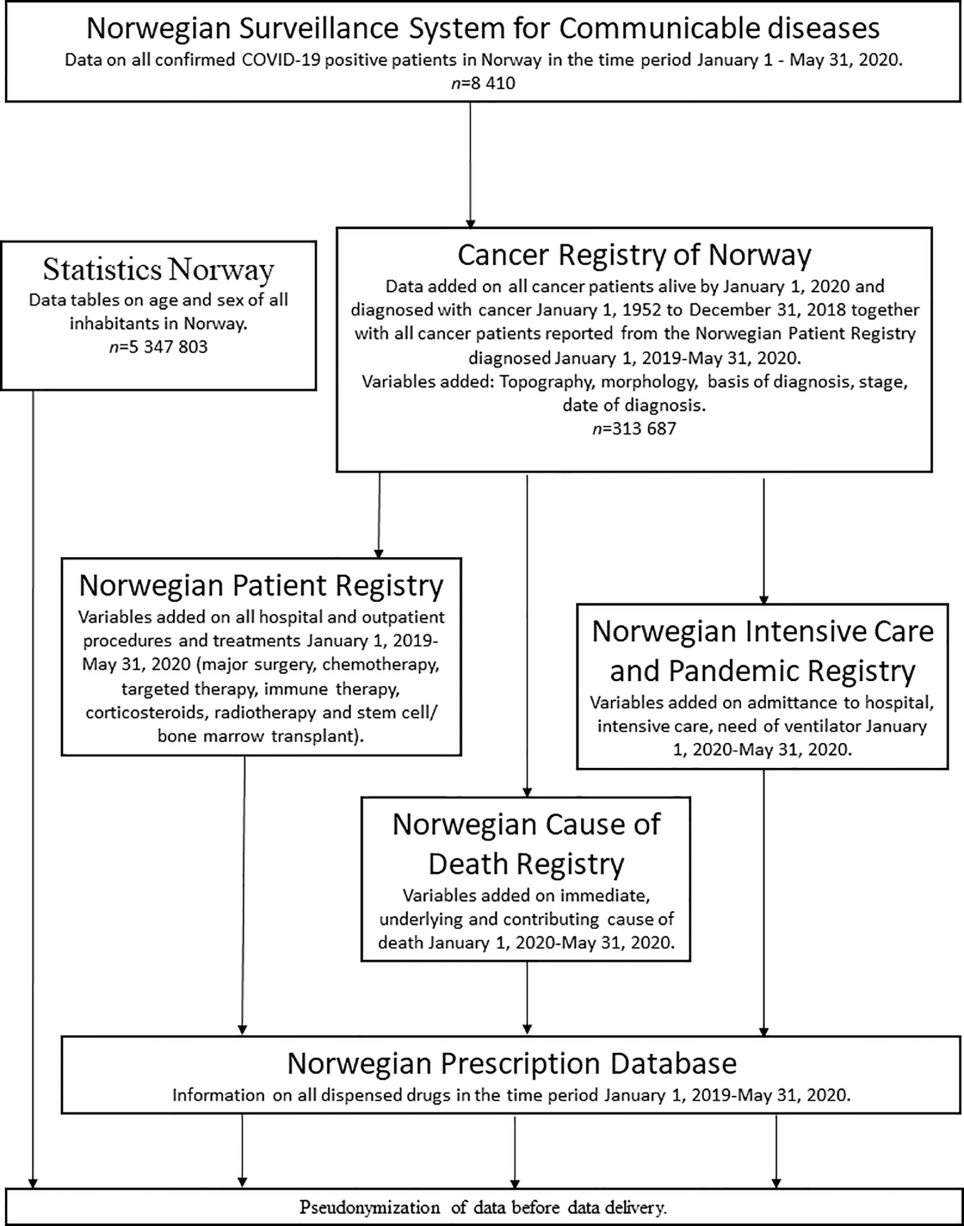
Flow-chart of data extraction and linkages.

We linked six registries in Norway with national coverage and documented high data quality. An overview of data extraction and linkages is shown in **Figure 1**. All registries are regulated according to the act relating to Personal Health Data Registries and established by the Norwegian Government. The registries are not based on consent, there is compulsory reporting and they all contain personal identifiable information.

Linkages were performed using the national identity number assigned to all citizens at birth or immigration.

Data on all confirmed COVID-19 positive patients were extracted from the Norwegian Surveillance System for Communicable diseases (MSIS), which is a registry based on mandatory electronic reporting from microbiological laboratories. In addition to a confirmed SARS-CoV-2 infection, data variables included for this study were: date(s) of test(s), result(s), age, sex, information about hospitalization and diagnosis.

Data on cancer occurrence was extracted from the Cancer Registry of Norway (CRN). We obtained data on all patients diagnosed with cancer from January 1, 1952 to December 31, 2018. Because the 2019 data were not available when the dataset was obtained, we also used data from the Norwegian Patient Registry (NPR) on patients diagnosed in the time period January 1, 2019-May 31, 2020. Patients diagnosed with cancer on the same date or later than the confirmed diagnosis of COVID-19 (23 patients) were excluded from the analysis.

Stage of disease in solid-cancers was defined as follows in the data from the CRN: localized to the primary tumor site, regional spread or distant metastases. If the patient later in NPR data was recorded with ICD-10 codes indicating advanced stage (secondary malignant neoplasms C77-C79) at hospitalization or admittance, this was defined as distant stage. Patients registered solely in NPR with ICD-10 codes C77-C79 were registered as having distant metastases. This was also done if patients were admitted for COVID-19 and were recorded in the Norwegian Intensive Care and Pandemic Registry data (NIPaR) as having distant metastases.

Procedure codes are reported from all hospitals to the National Patient Registry (NPR) for reimbursement purposes. We obtained data on all registered procedures irrespective of diagnosis for the period January 1, 2019-May 31, 2020. Time periods were defined as time between date of procedure and either date of confirmed SARS-CoV-2 infection or May 31, 2020. We included information on the following treatments during the past three months: major surgery, chemotherapy, targeted therapy, immune therapy, corticosteroids and radiotherapy. We included information on stem cell or bone marrow transplant if done within the past 12 months.

For cancer patients treatment was defined as follows: We defined major surgery as any procedure related to major organs (surgical procedures codes starting with AA, AB, BA, BB, BC, F,G,H, J,K,L) (19). Medical oncological procedures using chemotherapy or other medications, such as targeted therapy or immunotherapy (procedure codes starting with WBOC) (19). Radiotherapy included external radiotherapy, brachytherapy, proton treatment (procedure codes WEOA, WEOB, WEOC, AAG50, AAG60). Stem cell or bone marrow transplant (procedure codes RAGG15, RAGG20, RAGG25, RAGG30, RAGG35, RAGG40, RAGG50, RAGG55, RAGG60, RAGG65) (19).

All prescriptions payed for and administered by hospitals were available from NPR and were defined by the Anatomical Therapeutic Chemical (ATC) Classification System (20): Chemotherapy: All ATC codes starting with: L01A, L01B, L01C, L01D, L01XA, L01XX, L01XY. Targeted therapy: All ATC codes starting with L01XE, L01XC-minus immunotherapy codes. Immunotherapy (PD-1, PD-L1, CTLA-4): ATC codes: L01XC11, L01XC17, L01XC18, L01XC32, L01XC28, L01XC31. Corticosteroids: ATC codes starting with H02.

We obtained information on all prescribed drugs for all patients for the time period January 1, 2019-May 31, 2020 from the Norwegian Prescription Database (NorPD). All pharmacies in Norway register prescriptions electronically, and the information is forwarded to NorPD. The same ATC codes were used as for NPR data in defining treatment.

From the Norwegian Cause of Death Registry all deaths up to May 31, 2020 were included. The registry follows the WHO guidelines for certifying COVID-19 as a cause of death and the ICD-10 code U07.1 was used for defining COVID-19 as the underlying cause of death. The definition of the causal sequence leading to death due to COVID-19 was followed and 19 patients with COVID-19 as contributing but not underlying cause of death were excluded. Additional variables available were place of death (at home, nursery home/hospice or hospital).

From the Norwegian Intensive Care and Pandemic Registry we retrieved data on patients admitted to hospital or intensive care. Only patients with COVID-19 stated as cause of admission were included. Available variables were whether the patient needed intensive care and mechanical ventilation as well as number of days treated.

Information on age and sex distributions of the entire population were available from Statistics Norway.

### Ethical Approval

All registries used in this study have compulsory reporting and legal permission to collect and deliver data without the need to seek consent. The Committee for Medical and Health Research Ethics approved all aspects of the study (REK Midt, ref no 136767). In the last step of the data linkage, the patient’s national identification number was replaced by a unique pseudonym by Statistics Norway. All dates and times were replaced by values according to a set reference date.

### Statistical Analyses

Age standardized cumulative rates were calculated for COVID-19 (**Table 1**) and death due to COVID-19 (**Table 3**). Odds ratios were estimated as measures of relative risk using logistic regression. The outcomes in the regression models were death due to COVID-19 (**Table 4**) and the combined outcome death and/or admittance to hospital due to COVID-19 (**Table 5**). Two models were applied in the logistic regressions: an unadjusted univariate model and a multivariate model adjusted for age and gender. Multivariate risk ratios by Poisson regression gave similar results as odds ratios. Age adjustment was done according to age groups 0–29 and 5 year intervals to 100+. Odds ratios of COVID-19 among cancer patients compared to the non-cancer population were calculated by various demographic factors such as tumor subtype, time since diagnosis and treatment (**Table 2**).

**Table 1:** Age-standardized cumulative rates of COVID-19 among the cancer population versus the non-cancer population (per 100 000).

	COVID-19 rate in population with cancer	COVID- 19 rate in the population without cancer	COVID-19 rate ratio (cancer patients/patients without cancer)
**Total**	150.85 (122.11–179.59)	156.15 (152.68–159.62)	0.97 (0.78–1.19)
**Females**	163.13 (113.70–212.56)	157.90 (152.97–162.83)	1.03 (0.74–1.45)
**Males**	139.44 (105.66–173.21)	155.83 (150.81–160.85)	0.89 (0.69–1.16)
**0–49 years**	86.20 (58.42–113.98)	90.12 (87.53–92.71)	0.96 (0.68–1.35)
**50–69 years**	46.67 (39.60–53.73)	46.89 (45.00–48.78)	1.00 (0.82–1.21)
**≥70 years**	17.98 (15.90–20.07)	19.14 (17.81–20.47)	0.94 (0.78–1.13)

## Results

In the time period from January 1, 2020 to 31 May, 2020 a total of 8 410 patients were diagnosed with a COVID-19 infection in Norway. Of these, 547 (6.5%) were cancer patients. The proportion of individuals alive in the population and with a prevalent cancer by January 1, 2020 was 5.7% (305 846/5 347 873).

The cumulative rate of COVID-19 disease during the study period was similar in cancer patients and the rest of the population (**Table 1**). Although the rate was slightly lower among male cancer patients than in the male population without cancer, this was not statistically significant. There was a trend toward decreasing rate of COVID-19 with increasing age both in the population with cancer and the population without cancer and there was similar rate-ratios between cancer patients and patients without cancer.

Unadjusted odds ratios of COVID-19 among cancer patients compared to COVID-19 in the non-cancer population by tumor subtype and oncological treatment modality are shown in **Table 2**. Compared to the non-cancer population, there was a significant increased risk of COVID-19 in cancer patients, both for patients with solid and non-solid tumors. Also there was a significant increased risk of COVID-19 in patients with melanoma and thyroid malignancies. Patients diagnosed with cancer more than one year earlier appeared to be at increased risk in addition to cancer patients having undergone major surgery within the past 3 months. We did not find any increased OR related to different oncological treatment modality. There were no COVID-19 cases among patients having performed stem cell or bone marrow transplant within the last year.

**Table 2:** Unadjusted odds ratios (ORs) of COVID-19 among cancer patients compared to COVID-19 in the non-cancer population by tumor subtype and oncological treatment modality.

	Number of individuals with cancer and COVID-19	Number of individuals with cancer without COVID-19	Odds ratio of COVID-19 disease (95% CI)
**All cancer C00-96 (ICD-10)**	547	305299	1.15 (1.05–1.26)
**Solid tumors (all except C81–96)**	493	279686	1.13 (1.03–1.24)
**Non-solid tumors C81–96**	54	25613	1.35 (1.02–1.77)
**Digestive organs C15–26**	73	40671	1.15 (0.90–1.45)
**Respiratory organs C30–34, C38**	13	9986	0.84 (0.44–1.43)
**Melanoma of the skin C43**	65	27071	1.54 (1.19–1.97)
**Skin, non-melanoma C44**	33	14932	1.42 (0.98–2.00)
**Breast C50**	85	49738	1.10 (0.88–1.36)
**Female genital organs C51–58**	33	23827	0.87 (0.60–1.23)
**Male genital organs C60–63**	105	58874	1.16 (0.95–1.42)
**Urinary organs C64–68**	35	20852	1.08 (0.75–1.50)
**Central nervous system C70–72**	20	13685	0.94 (0.57–1.45)
**Thyroid gland C73**	20	5975	2.15 (1.31–3.32)
**<1 year since cancer diagnosis**	53	38806	0.88 (0.66–1.15)
**1–5 years since cancer diagnosis**	151	79152	1.22 (1.04–1.44)
**≥5 years since cancer diagnosis**	343	187341	1.18 (1.05–1.31)
**Chemotherapy including targeted therapy and immunotherapy last 3 months**	71	45599	1.00 (0.78–1.26)
**Surgery last 3 months**	90	44566	1.30 (1.04–1.60)
**Stem cell or bone marrow transplant last 12 months**	0	514	0.00 (0.00–4.80)
**Radiotherapy last 3 months**	7	4929	0.91 (0.37–1.88)

The number of individuals without cancer but with COVID-19 was 7 841 and the number of individuals without cancer and without COVID-19 was 5 034 186.

In the entire population, there were a total of 214 deaths due to COVID-19 and of these 56 patients had a cancer diagnosis. Age-standardized rates of death due to COVID-19 among all cancer patients together with rates of death among the whole non-cancer population are shown in **Table 3**. A non-significant trend of lower rate among cancer patients was found, especially in males.

**Table 3:** Age-standardized cumulative rates of death due to COVID-19 among all cancer patients compared to the non-cancer population (per 100 000).

	Rate of death due to COVID-19 in the cancer population	Rate of death due to COVID-19 in the non-cancer population	Rate ratio of death due to COVID-19 in cancer patients compared to the non-cancer population
**Females**	3.07 (1.90–4.24)	2.84 (2.17–3.51)	1.08 (0.58–2.02)
**Males**	4.08 (2.23–5.93)	6.09 (4.75–7.43)	0.67 (0.35–1.27)
**Total**	3.55 (2.52–4.58)	4.05 (3.41–4.69)	0.88 (0.56–1.36)

The fatality rate of COVID-19 among cancer patients was 0.10 with an univariate OR of death of 5.55 (4.03–7.62) **Table 4**. However, when adjusting for age and sex this increase was non-significant compared to the patients without cancer. There was no significant difference in ORs of death for patients with solid tumors or non-solid tumors by multivariate analysis compared to the population without cancer. Patients with distant metastases had a fatality rate of 0.35 and there was a significant increased OR of death by multivariate analysis of 9.31 (2.60–33.34). No other factors such as time since cancer diagnosis, cancer treatment or admission for COVID-19, significantly increased ORs of death by multivariate analysis.

**Table 4:** Fatality rates and odds ratios of death due to COVID-19 among cancer patients compared to the rate of death due to COVID-19 among the population without cancer.

	Died of COVID-19	With COVID-19	COVID-19 fatality rate	Univariate Odds ratio (95% CI)	Multivariate Odds ratio (95% CI)
**Individuals without cancer and with COVID-19**	158	7 841	0.02	1.00 (.–.)	1.00 (.–.)
**All cancer C00-96 (ICD-10)**	56	547	0.10	5.55 (4.03–7.62)	0.99 (0.68–1.42)
**Solid tumors (all except C81–96)**	53	493	0.11	5.86 (4.23–8.11)	0.99 (0.68–1.44)
**Non-solid tumors C81–96**	3	54	0.06	2.86 (0.88–9.26)	1.00 (0.28–3.65)
**Localized/regional disease**	36	367	0.10	5.29 (3.62–7.72)	0.88 (0.57–1.35)
**Distant disease**	6	17	0.35	26.52 (9.69–72.61)	9.31 (2.60–33.34)
**Unknown stage**	14	163	0.09	4.57 (2.58–8.08)	0.85 (0.44–1.63)

The proportion admitted to hospital due to COVID-19 among cancer patients was 120/547 (21.9%) versus 755/7841 (9.6%) among patients without cancer. The proportion treated in ICU was 17/547 (3.1%) for cancer patients versus 157/7841 (2.0%) in non-cancer patients and proportions treated with mechanical ventilator were 17/547 (3.1%) and 135/7841 (1.7%), respectively. Rates of death due to COVID-19 combined with admission to hospital due to COVID-19 are shown in **Table 5**. Significant increased ORs for death and/or admission due to COVID-19 was seen for cancer patients diagnosed <1 year before COVID-19 diagnosis (OR 2.08 CI 1.14–3.80). Significant increase was also found for chemotherapy including targeted therapy and immunotherapy last 3 months (OR 1.80 CI 1.07–3.01) and also for those treated the last 12 months (OR 1.63 CI 1.05-2.52). Significant increase was also found for patients who had recent surgery (OR 2.19 CI 1.40–3.44).

**Table 5:** Rates of death due to COVID-19 and/or admission to hospital due to COVID-19.

	Cancer patients died or admitted to hospital because of COVID-19	Cancer cases	COVID-19 fatality/admittance rate	Univariate Odds ratio (95% CI)	Multivariate Odds ratio (95% CI)
**Cancer free population**	854	7 841	0.11	1.00 (.–.)	. (.–.)
**All cancer C00-96 (ICD-10)**	160	547	0.29	3.38 (2.78–4.12)	1.14 (0.91–1.42)
**Solid tumors**	150	493	0.30	3.58 (2.92–4.39)	1.18 (0.93–1.48)
**Non-solid tumors C81–96**	10	54	0.19	1.86 (0.93–3.71)	0.84 (0.41–1.74)
**Localized/regional disease**	106	367	0.29	3.32 (2.62–4.21)	1.09 (0.84–1.42)
**Distant disease**	8	17	0.47	7.27 (2.80–18.90)	2.62 (0.94–7.28)
**Unknown stage**	46	163	0.28	3.22 (2.27–4.56)	1.16 (0.79–1.69)
**<1 year since cancer diagnosis**	21	53	0.40	5.37 (3.08–9.35)	2.08 (1.14–3.80)
**1–5 years since cancer diagnosis**	40	151	0.26	2.95 (2.04–4.26)	1.11 (0.74–1.65)
**≥5 years since cancer diagnosis**	99	343	0.29	3.32 (2.60–4.24)	1.04 (0.79–1.37)
**Chemotherapy including targeted therapy and immunotherapy last 3 months**	28	71	0.39	5.33 (3.29–8.62)	1.80 (1.07–3.01)
**Immunotherapy last 3 months**	6	15	0.40	5.45 (1.94–15.36)	1.63 (0.55–4.81)
**Corticosteroids last 3 months**	19	45	0.42	5.98 (3.30–10.85)	1.70 (0.91–3.20)
**Radiotherapy last 3 months**	3	7	0.43	6.14 (1.37–27.46)	2.67 (0.54–13.14)
**Surgery last 3 months**	42	90	0.47	7.16 (4.70–10.90)	2.19 (1.40–3.44)

Results are shown for cancer patients compared to those who died or were admitted due to COVID-19 among the population without cancer.

A total of 32.1% of cancer patients with SARS-CoV-2 died in hospital compared to 40.5% of those without cancer. Similar proportions for nursing homes/hospice were 64.3% among cancer patients compared to 57% of those without cancer.

## Discussion

Cancer patients have been identified as a group with higher risk of a more adverse outcome of SARS-CoV-2 -infection than those without cancer (2, 3, 21). Cancer patients often have comorbidities increasing the susceptibility further. Cancer patients with COVID-19 commonly present with symptoms similar to the general population such as fever and dry cough with dyspnoea and fatigue possibly more frequent (3, 22). Additional findings such as anemia and hypoproteinemia may be found (22). Severe anoxia and more rapid progress may be seen possibly due to myelosuppression and an immunosuppressive state due to malignancy and antitumor therapy which may include surgical, pharmacological and radiation therapies (23).

We did not find a significant increased risk of dying from COVID-19 among cancer patients overall but patients with distant metastases had a significant increased OR of death compared to the rest of the population. When combining death and/or admission due to COVID-19 as a parameter of serious outcome we found significantly increased risk of SARS-CoV-2 -infection among cancer patients diagnosed <1 year since cancer diagnosis and for patients undergoing surgery or treated with systemic cancer therapy the last 12 and 3 months.

Early reports from China reported that cancer patients were overrepresented among patients with COVID-19 (24, 25). Hospital admission and recurrent visits were proposed as risk factors for infection, but numbers were small. Later reports on the prevalence of cancer COVID-19 patients have varied, and recent studies have shown proportions of cancer patients ranging 0.4-8% among confirmed COVID-19 cases (6, 11, 12, 26–30).

This study was limited to the first months of the pandemic and we found that the risk of COVID-19 disease among cancer patients was similar to the general population. Higher rates in male cancer patients have previously been reported (5) but we found a non-significant tendency toward a lower rate in male cancer patients compared to females. The trend of decreasing rate of COVID-19 with increasing age both in the population with cancer and the population without cancer may be due to recommended measures early in the pandemic, especially in patients with several risk factors and with a focus on high age (31).

In line with previous reports we found that patients with leukemia and lymphomas together with endocrine tumors were at greater risk of COVID-19 (5, 6) while we did not find this for patients with lung cancer.

Increased fatality rate in cancer patients with COVID-19 has been found in several studies (5, 6, 9, 12, 32). We found that the death rate among cancer patients was comparable and non-significantly lower in both females and males compared to the non-cancer population when adjusting for age. Significantly increased risk of death due to COVID-19 was found for patients with distant disease with a fatality rate of 0.35 with a significant increased OR of 9.31. However, numbers were small.

Increased COVID-19 attributable mortality reported in hematological malignancies (5, 9, 32) was not found in this study and in line with other publications (6) while in contrast to others (11).

Previous reports have suggested that high mortality from COVID-19 in cancer patients appears to be driven by age, gender, and comorbidities (6, 14). Cancer patients are in general at high age with coexisting chronic diseases and several reports do not adjust for known risk factors such as age and gender when interpreting results (5, 11). In a multivariate model, we found that risk of death in cancer patients with COVID-19 did not differ from the risk of death in non-cancer patients with COVID-19.

Subgroups of cancer patients with high risk of severe outcome including deaths should be prioritized for vaccination. In contrast to a previous report showing significant excess mortality risk from recent chemotherapy (32) we found that recent cancer therapy did not significantly influence mortality when adjusted for age and sex. This is in line with previous reports that do not confirm increased mortality attributable to recent treatment (6, 14, 15). An increased risk of COVID-19 illness severity caused by targeted immunotherapy has been reported (16) and in this study the increase in risk for the combined outcome of death and/or admittance to hospital due to COVID-19 was non-significant after adjustment. We found significant increased risk of severe outcome for cancer patients diagnosed <1 year since cancer diagnosis, and systemic cancer treatment the last 3 and 12 months.

Strengths of this study include high quality data from registries including specifically completeness of all confirmed COVID-19 cases, cancer diagnosis from the CRN, treatment, intensive care data and reliability of causes of death. Weaknesses include low general total count of COVID-19 cases with resulting wide confidence intervals. Reported case counts and results will vary depending on availability of testing, and often underestimate the burden in general of COVID-19. Asymptomatic carriers may have been missed, together with patients with mild symptoms not tested and patients who died with unrecognized COVID-19.

In conclusion we found similar age adjusted rates of COVID-19 in the population with cancer as in the population without cancer. Unadjusted analysis showed that patients having undergone major surgery within the past 3 months had an increased risk while we did not find any increased OR related to other oncological treatment modalities. For cancer patients in general with COVID-19, there was no increased risk of death compared to the patients without cancer. However we did find an increased risk for patients with metastatic disease. For the combined outcome serious COVID-19 disease (death and/or admittance to hospital), we found an increased risk for patients with recent cancer diagnosis (<1 year prior to infection) and for patients receiving ongoing or recent treatment. We suggest those subgroups of cancer patients, i.e. metastatic disease, ongoing/recent therapy or recent diagnosis, to be prioritized in national vaccination programs. Otherwise, our results suggest that the large majority of those living with a cancer diagnosis appear not to be at increased risk of COVID-19 infection or serious disease.

## Data Availability Statement

The raw data supporting the conclusions of this article will be made available by the authors, without undue reservation.

## Ethics Statement

The study was reviewed and approved by the Regional Committees for Medical and Health Research Ethics REK Midt, ref no 136767. Written informed consent from the participants was not required in accordance with national legislation and institutional requirements.

## Author Contributions

Substantial contribution to the conception and design of the work, including acquisition, analysis, interpretation of data; drafting and revising the manuscript and approving the submitted version (TJ, SS, SA, EB, GU, AS, ÅH). All authors contributed to the article and approved the submitted version.

## Funding

Solely own institutional funding

## Conflict of Interest

The authors declare that the research was conducted in the absence of any commercial or financial relationships that could be construed as a potential conflict of interest.
